# A first exploratory comparison of the behaviour of wolves (*Canis lupus*) and wolf-dog hybrids in captivity

**DOI:** 10.1007/s10071-024-01849-7

**Published:** 2024-03-02

**Authors:** Federica Amici, Simone Meacci, Emmeline Caray, Linda Oña, Katja Liebal, Paolo Ciucci

**Affiliations:** 1 Life Sciences, Institute for Biology, Human Biology and Primate Cognition, Leipzig University, Leipzig, Germany; 2 Department of Comparative Cultural Psychology, Max Planck Institute for Evolutionary Anthropology, Leipzig, Germany; 3 Department of Biology and Biotechnology “Charles Darwin”, Sapienza University of Rome, Rome, Italy; 4 Department of Life Sciences, University of Strasbourg, Strasbourg, France

**Keywords:** Hybridization, Introgression, Neophobia, Reaction to humans, Social networks

## Abstract

Extensive introgression of genes from domesticated taxa may be a serious threat for the genomic integrity and adaptability of wild populations. Grey wolves (*Canis lupus*) are especially vulnerable to this phenomenon, but there are no studies yet assessing the potential behavioural effects of dog-introgression in wolves. In this study, we conducted a first systematic comparison of admixed (N = 11) and non-admixed (N = 14) wolves in captivity, focusing on their reaction to unfamiliar humans and novel objects, and the cohesiveness of their social groups. When exposed to unfamiliar humans in the experimental task, wolves were more vigilant, fearful and aggressive than admixed wolves, and less likely to approach humans, but also more likely to spend time in human proximity. When exposed to novel objects, wolves were more aggressive than admixed wolves, less likely to spend time in object proximity, and more likely to interact with objects, but also less vigilant and as fearful as admixed wolves. Finally, social networks were more cohesive in wolves than in admixed wolves. Although caution is needed when comparing groups of captive individuals with different life experiences, our study suggests that dog admixture may lead to important behavioural changes in wolves, with possible implications for conservation strategies.

## Introduction

Hybridization is a natural process that can lead to the permanent transfer of genetic information (i.e. introgression) between species or genetically differentiated populations, and often results in positive evolutionary outcomes (e.g. genetic enrichment, evolutionary novelties; Arnold 1997; Brennan et al. 2014). However, hybridization can also be favoured by human impact and interference (i.e. anthropogenic hybridization), for instance through habitat loss or fragmentation or the introduction of exotic or domestic species, which may cause abnormal sympatry (Allendorf et al. 2001; Rhymer and Simberloff 1996). Extensive introgression of genes from domesticated taxa, in particular, may be a serious threat for the genomic integrity and adaptability of wild populations (Allendorf et al. 2001), as domesticated taxa are artificially selected and the introgression of their gene variants into wild populations is usually maladaptive (Allendorf et al. 2013; Gottelli et al. 1994; Randi 2008; Simberloff 1996).

One species which is especially vulnerable to anthropogenic hybridization are grey wolves (*Canis lupus*), a taxon that plays a key ecological role (Ripple and Beschta 2004; Roemer et al. 2009). In evolutionary terms, divergence between wolves and domestic dogs (*Canis lupus familiaris*) is a relatively recent phenomenon (i.e., about 40 Kya, although still debated; Freedman et al. 2014) and reproductive isolation is not yet complete, so that hybridization may be rather common, especially in areas with high degrees of sympatry (Harrison and Larson 2014; Hindrikson et al. 2017; Pilot et al. 2021; Randi 2008; Wayne and Shaffer 2016). Although the extent of back-crossings and introgression of dog gene variants into wolf populations is still largely unknown, and probably is as old as domestication itself (Freedman et al. 2016; Pilot et al. 2018), several recent studies indicate that the phenomenon may be substantial and increasing in some European wolf populations (Hindrikson et al. 2012; Pilot et al. 2018, 2021; Stronen et al. 2012). Genetic surveys at the local scale indicate that the proportion of admixed individuals in wolf populations can be as high as 50–70% in human-modified landscapes (Salvatori et al. 2020; Santostasi et al. 2021), currently making wolf-dog hybridization one of the most relevant threats for wolves in Europe (Hindrikson et al. 2012).

Expectedly, introgression of dog genes in the wolf genome may lead to important phenotypic changes, including morphological, physiological, and behavioural ones. Indeed, several anomalous morphological traits have been documented in wolf-dog hybrids and introgressed individuals (Ciucci 2012; Galaverni et al. 2017; Lorenzini et al. 2013), including traits with a genetic origin like dewclaws on hind legs, depigmented claws, and melanistic coat (Caniglia et al. 2013; Ciucci et al. 2003; Hedrick 2009). Anomalous physiological traits linked to the introgression of dog genes include different reproductive phenology in captive admixed wolves (Iljin 1941), and perhaps in wild ones (Crispino et al. 2021). In contrast, there is to date a surprising lack of information about behavioural changes in wolves that might be linked to the introgression of dog gene variants (vonHoldt et al. 2017). Yet, recent genomic work suggests that even minimal levels of dog-introgression can have important effects on brain function and behaviour in wolves (see e.g. Pilot et al. 2021, on the introgression of four dog gene variants involved in neurotransmission and neurodevelopment). Understanding whether and how dog admixture is linked to behavioural changes in wild wolves is crucial, because these changes may affect their life history traits with effects on social interactions within and among packs, predatory behavior, pack size, dynamics, reproduction and density-dependent mechanisms such as dispersal (Newsome et al. 2017; Sparkman et al. 2012). Moreover, behavioural changes may affect wolves’ relationship with other species, including the frequency and nature of interactions with humans. The latter is particularly relevant management-wise, as variation in the behaviour of admixed wolves towards humans may add to the inherent complexity of managing wolf-human relationships, including human fear of wolves (Linnell and Alleau 2016), livestock depredation by wolves (Kaartinen et al. 2009), predation on dogs (Kojola and Kuittinen 2002) and dependency on anthropogenic food sources (Newsome et al. 2016).

To date, there are no studies, to our knowledge, assessing the behavioural effects of dog-introgression in wolves. However, given the strong genetic basis of dog behaviour (e.g. Morrill et al. 2022; Salomons et al. 2021), and the underlying effects of artificial selection on the dog genome (Bergström et al. 2020; Freedman et al. 2016), it is reasonable to expect behavioural changes through the introgression of dog gene variants in wild wolf populations (Leonard et al. 2013). Through the dog domestication process, there was likely a selection for individuals that were more tolerant and/or attracted to humans, and that showed lower levels of aggression and fear toward humans and their environment (Hare et al. 2002; Li et al. 2013; vonHoldt and Driscoll 2016). This has at least three implications. First, as confirmed by several studies, dogs may be less fearful and aggressive, more likely to approach and interact with humans, and perhaps better able to use human cues and understand their communicative intentions, as compared to wolves (e.g., Hare et al. 2002; Lazzaroni et al. 2020; Miklósi et al. 2003; Oláh et al. 2021; but see Range et al. 2019). Crucially, these differences hold true also when comparing dogs and wolves having received a similar exposure to humans during development (Salomons et al. 2021), suggesting a genetic basis of these behavioural differences. Recent studies, indeed, confirm the existence of structural changes in specific genomic regions of dogs, which are linked to hypersocial behaviour (i.e. exaggerated propensity to initiate social contact), and likely explain increased tolerance and attraction to humans (vonHoldt et al. 2017). Second, as a result of the domestication process, dogs have been experimentally found to be less fearful, less aggressive, and more likely to approach novel anthropogenic objects, as compared to wolves (e.g., Fritts et al. 2003; Hare and Tomasello 2005; Kaulfuβ and Mills 2008; Klinghammer and Goodmann 1987; Kniowski 2012; Zimen 1987), even if controlling for their previous exposure to humans (Salomons et al. 2021). However, free-ranging feral dogs are also less defensive of their territory than wolves (Boitani and Ciucci 1995; Boitani et al. 2017), and this might be linked to a reduction in their explorative tendencies and persistence in interacting with novel objects (Moretti et al. 2015). Indeed, as compared to wolves, dogs are usually less fearful, but also less motivated to explore novel objects (e.g., Frank and Frank 1985; Moretti et al. 2015). Third, increased attraction towards humans may result in looser interactions with conspecifics. Even when free-ranging, dogs are usually less likely than wolves to form strong bonds and cohesive groups with their conspecifics, and are considered to be facultatively social (Boitani and Ciucci 1995; Boitani et al. 2007; van Kerkhove 2004), being often solitary or forming more temporary associations (Beck 1973, 1975; Berman and Dunbar 1983; Daniels 1983; Daniels and Bekoff 1989; Ortolani et al. 2009; Rubin and Beck 1982; see Bonanni and Cafazzo 2014). In contrast, wolves usually live in highly cohesive social groups and form strong social bonds with each other (e.g., Cordoni and Palagi 2019; Mech 1999; Mech and Boitani 2003; Packard 2012), which are thought to promote cohesion among group members and cooperation during group activities, like hunting (MacNulty et al. 2009, 2012; Mech 1975), breeding (Mech 1999; Packard et al. 1992), and territorial defence (Harrington and Mech 1979; Mech 1993; Mech and Boitani 2003).

If the above behavioural differences between wolves and dogs have a genetic basis (see Salomons et al. 2021), it is possible that the introgression of dog genes may trigger behavioural changes in admixed wolves (Leonard et al. 2013). However, these changes may be too subtle to be captured with the methods that are traditionally used to study wolves in the wild (e.g. telemetry), where ethological observations and experimental approaches are not yet fully viable. In this study, we therefore conducted a first systematic comparison of admixed and non-admixed wolves in captivity, focusing on their reaction to unfamiliar humans and novel objects, and the cohesiveness of their social groups. We focused on these behaviours, because they are known to differ between dogs and wolves (Boitani and Ciucci 1995; Fritts et al. 2003; Hare and Tomasello 2005; Hare et al. 2002; Kaulfuβ and Mills 2008; Klinghammer and Goodmann 1987; Kniowski 2012; Lazzaroni et al. 2020; Miklósi et al. 2003; Oláh et al. 2021; Zimen 1987) and might therefore be affected by the introgression of dog genes. We hypothesized that dog admixture may trigger differences in individuals’ reaction to unfamiliar humans and novel objects, and in the cohesiveness of their social networks (Table 1). First, we predicted that, when exposed to unfamiliar humans, non-admixed wolves (hereafter, wolves) would be more neophobic (i.e. more vigilant, more fearful, more aggressive and less likely to spend time in proximity) than admixed wolves, and less likely to interact with them (Prediction 1). Second, we predicted that, when exposed to novel objects, wolves would be more neophobic than admixed wolves, but also more likely to interact with the stimuli, being more explorative because of their more pronounced territorial attitude (Prediction 2). Finally, we predicted that wolves would be characterized by more cohesive social networks (i.e. with higher densities; see Farine and Whitehead 2015), as compared to admixed wolves (Prediction 3).

**Table 1:** Predictions of the study comparing behavioural responses in wolves (W) and admixed wolves (AW) in captivity

Predictions		Confirmed?
1	When exposed to unfamiliar humans, as compared to AW…	
	(a) W are more likely to be vigilant	Yes (M1)
	(b) W are more likely to show fearful behaviour	Yes (only W)
	(c) W are more likely to show aggressive behaviour	Yes (only W)
	(d) W spend less time in human proximity	No* (M2)
	(e) W are less likely to be explorative	Yes (only AW)
2	When exposed to novel objects, as compared to AW…	
	(a) W are more likely to be vigilant	No* (M3)
	(b) W are more likely to show fearful behaviour	No (M4)
	(c) W are more likely to show aggressive behaviour	Yes (only W)
	(d) W spend less time in object proximity	Yes (M5)
	(e) W are more likely to be explorative	Yes (M6)
3	W have more cohesive social networks than AW	Largely (SNA)

The last column shows whether the predictions were confirmed, and how they were tested (M1 to M6 stand for the generalized linear mixed models run, SNA stands for social network analyses, and “only W/AW” means that only wolves/admixed wolves showed the behaviour; the asterisk refers to results that were opposite to the predictions; see Table 3 for actual measures)

## Methods

### Study subjects

We studied two groups of admixed wolves with known life experiences and genetic profile. All admixed wolves were backcrosses of first or second generation (BC1, BC2) that had been live captured in their natural habitats and moved in captivity as part of wolf conservation programs (Bocci et al. 2015). The two groups had never been studied before. We observed the first group of admixed wolves (AW1) in April and May 2022, the second group (AW2) in October and November 2022, and a group of wolves (W) from January to May 2022. All study subjects were individually recognized thanks to differences in their fur, body and face. Before starting the observations, we habituated the groups to the presence of the observer, who was always located outside the enclosure.

The first study group (AW1) included six admixed males hosted in the faunistic area of Riserva Naturale Regionale Lago di Penne, Italy (see Table 2). The individuals were 5-year old brothers that had been captured in 2017, in the Gran Sasso—Monti della Laga National Park, Italy, when they were about 20 days old. They were raised by human caregivers, first with bottle feeding, then with minced meat. When they were about three months old, they were relocated in their current enclosure of about half a hectare, which included an artificial pond and a grove. Human exposure was limited to the few visitors who could observe the group from a little observation area granting visual access to large part of the enclosure, and to the keepers feeding the group daily by leaving pellets and other food in one area of the enclosure. All individuals had been vasectomized, and were hormonally intact (Umberto Di Nicola, pers. comm).

**Table 2:** Details of the captive individuals we observed to contrast behaviour between a group of wolves (W) and two groups of admixed wolves (AW)

Study group	Subject	Sex	Age (years)	Rank
Admixed wolves (AW1)	Felpato	Male	5	1.00
	Ivan	Male	5	0.21
	Kevin	Male	5	0.18
	Licaone	Male	5	0.23
	Lucio	Male	5	0.00
	Mr Pickles	Male	5	0.52
Admixed wolves (AW2)	Bing	Female		
	Explorer	Female	8	0.00
	Firefox	Female	8	1.00
	Safari	Female		0.94
	Yahoo	Female	8	
Non-admixed wolves (W)	Angel	Female	2–3	0.45
	Anonymous	Female	2–3	0.63
	Eleven	Female	2–3	0.00
	Eren	Male	5	1.00
	Jackal	Male	2–3	0.51
	Jon Snow	Male	5	0.66
	Kaz	Male	5	0.55
	Lagerta	Female	2–3	0.46
	Mikasa	Female	9	0.88
	Mr grey	Female	2–3	0.43
	Noname	Female	2–3	0.68
	None	Female	2–3	0.40
	Ragnar	Male	2–3	0.63
	Witcher	Female	2–3	0.24

Empty cells represent unknown values. Rank values range from 0 to 1 depending on the outcome of dyadic agonistic interactions (see text)

The second group (AW2) included five admixed females housed at the Parco Faunistico del Monte Amiata, Italy (see Table 2). These admixed wolves were also live captured from the wild for conservation purposes and came from different litters. After being housed for 7–9 years in a smaller enclosure, they were moved to their current location one month before the start of our research. Three eight-year-old sisters (i.e., Explorer, Firefox, Yahoo) were captured in 2014 in the surroundings of Siena, Italy, when they were about two months old, and were raised by human caregivers in a recovery centre in Semproniano, Italy, where they were hosted together until the transfer to their current location. Safari was captured in proximity of Firenze, Italy, and Bing in Scansano, Italy, both in 2016. Their enclosure in Monte Amiata was about nine hectares and consisted of a grass clearing on top of a hill bordered by woods. A long corridor allowed visitors to reach an observation turret, from which it was possible to see part of the enclosure. Therefore, subjects were not always visible (Bing and Yahoo never appeared to the observer). Exposure to humans was limited to the few visitors and the keepers, who fed them daily by placing pieces of meat close to the observation turret to facilitate sightings. All individuals had been sterilized via tubal ligation and were hormonally intact.

For comparison, we also studied a group (W) of 14 Hudson wolves (*Canis lupus hudsonicus*), a subspecies of the grey wolf, hosted at the Osnabrück Zoo, in Germany. At the onset of our study, the group included eight females and six males aged between 2 and 9 years: three male individuals from the same litter were born in the Amsterdam Zoo in the Netherlands, one female individual was born in the Olomouc Zoo in the Czech Republic, and 10 individuals (8 females and 2 males) were from two different litters that were born in the Osnabrück Zoo (see Table 2). Based on the Osnabrück Zoo studbook, all the individuals were classified as pure wolves, and none of them showed morphological peculiarities (e.g. melanistic coat, dewclaws on hind legs, depigmented claws) suggesting a misclassification by the zoo. The wolf enclosure measured 0.45 ha and was smaller than the ones where AW1 and AW2 lived. It was connected to the one where black bears live during their winter sleep, and then limited to a smaller area from spring. The enclosure included open woods with tall trees and a walkway running along the edges, which allowed a clear view on all individuals. Wolves were exposed to the zoo visitors and the keepers, who usually fed them every other day by placing dead chickens, rabbits and sometimes fawns in the enclosure. Our study groups were not bred and raised for the purpose of this study, and their living conditions and previous life experiences were therefore not identical. The study groups differed in terms of group size and composition, their previous life experiences, and the size of their enclosures. All the admixed wolves, in particular, spent the first weeks with their mother in the wild and after capture for management purposes were later raised by humans in captivity; differently, wolves were born and housed in a zoo, where they were regularly exposed to keepers and visitors. Capturing this variation with categorical descriptors was not possible, as differences in life experiences across individuals and groups were mostly graded. Therefore, the following comparisons between wolves and admixed wolves should be taken with caution, bearing in mind all the limitations further examined in the Discussion.

### Inter-observer reliability

For all study groups, we used the same experimental protocol, which included two experimental tasks (i.e., a human task and an object task, to assess individuals’ reaction to unfamiliar humans and novel objects), and behavioural observations to assess patterns of affiliative and agonistic interactions across study subjects. Two different experimenters collected data: SM in AW1 and AW2, and EC in W. Inter-observer reliability was ensured in three ways. First, the two experimenters trained together for inter-observer reliability before starting data collection, by collecting behavioural observations in W for approximately two weeks, until reaching 80% inter-observer reliability for the coded behaviours. Second, all experimental tasks were video-recorded and later coded by two observers (SM, EC or FA), and upon disagreement, the video was coded together and eventually also coded by the third observer, until reaching an agreement on all the measures to be included. Third, a coder naïve to the hypothesis re-coded a subset of the videos of the experimental tasks (i.e. 10% of the Human task, and 25% of the object task). Inter-observer reliability was good for all the variables coded both in the human task (i.e. Cohen’s k for the probability of showing vigilance in the first 10 s of the trial: k = 0.78, N = 30; for the probability of showing fearful behaviour toward humans: k = 0.65, N = 37; for the probability of showing aggressive behaviour: k = 1.00, N = 37; for the probability of exploring humans: k = 1.00, N = 37; and for the probability of moving closer/remaining at the same distance from humans: k = 1.00, N = 30; all *p* < 0.001) and in the object task (i.e. Cohen’s k for the probability of showing vigilance in the first 10 s of the trial: k = 0.76, N = 13, *p* = 0.005; for the probability of showing fearful behaviour toward objects: k = 1.00, N = 18, *p* < 0.001; for the probability of showing aggressive behaviour: k = 0.88, N = 18, *p* < 0.001; for the probability of exploring objects: k = 1.00, N = 18, *p* < 0.001; and Spearman’s correlation for the proportion of time spent in proximity: ρ = 0.94, N = 18, *p* < 0.001).

### Human task

The human task aimed to assess individuals’ reaction to unfamiliar humans. We adapted the procedure used in previous studies on other species (e.g., see Amici et al. 2020; Damerius et al. 2017). Trials lasted 5 min and started when an unfamiliar human, outside of the enclosure and well-visible to the group, walked with a steady pace towards a part of the enclosure usually inaccessible to visitors. Upon approaching the enclosure, the human stopped in front of the study subjects, looking in their direction. All trials were video-recorded, and later coded from the videos. We conducted 20 trials in the wolf group (W) and in one admixed group (AW1), but only 7 trials in AW2, for logistic reasons (i.e. to avoid prolonged interference with the daily routines of the keepers). To ensure that the reaction to the humans did not depend on the specific characteristics of the individual humans, we used four different humans for each study group, with their order being pseudo-randomized across trials. All humans were volunteers who were not familiar to the study subjects, and differed across study sites.

For each trial and visible individual, we first coded individual identity and trial number. We further coded whether the visible individual (i) showed vigilance in the first 10 s of the trial, (ii) showed fearful behaviour toward the human (i.e., showing submissive behaviours or fleeing), (iii) aggressive behaviour, or (iv) exploration of the human (i.e., approaching within 5 m and sniffing without fearful or aggressive behaviour), at least once during the trial. Finally, we coded (v) whether visible individuals moved closer/remained at the same initial distance from the human or moved further, in the first 10 s of the trial. Time was always measured in seconds using a chronometer, while positions and distances were approximated in meters.

### Object task

The object task aimed to assess individuals’ reaction to novel objects. We adapted the procedure used in previous studies on wolves and dogs (e.g., Marshall-Pescini et al. 2017; Moretti et al. 2015). Trials lasted 20 min and started when the novel object was introduced in the enclosure, in an area frequently visited by group members. All trials were video-recorded, and later coded from the videos. In each group, we conducted four trials (with an interval of at least three days between two consecutive trials), alternating two different objects (i.e., a blue plastic inflatable ball with a diameter of approximately 70 cm and a yellow kettlebell of approximately 4 kg) to reduce individuals’ habituation to the object.

For each trial and visible individual, we first coded individual identity, object used (i.e., ball or kettlebell), trial number, time duration in which the individual was visible during the trial (hereafter, observational effort), and initial position of the individual relative to the object. We further coded whether visible individuals (i) showed vigilance in the first 10 s of the trial, (ii) showed fearful behaviour toward the object (i.e., showing submissive behaviours or fleeing), (iii) aggressive behaviour, or (iv) exploration of the object (i.e., approaching the object within 5 m, and sniffing, touching or playing with it, without fearful or aggressive behaviour), at least once during the trial. Finally, we coded (v) the proportion of time spent in proximity (i.e. closer/at the same initial distance to the object), out of the individual observational effort. Time was measured in seconds and positions and distances approximated in meters, as above.

### Behavioural observations

We conducted 20-min focal observations (Altmann 1974) on all the individuals of the study groups (except for Bing and Yahoo in AW2 that were never visible). Focal animals were selected in a pseudo-randomized order to ensure a similar number of focal samples across subjects, and were observed using the app Cybertracker (version 1.0.415). To assess affiliative interactions, during the focal observations we recorded as continuous measures the time spent in 2 m-proximity, social play and grooming, also specifying partner identity. To assess agonistic interactions and dominance hierarchies, we recorded ad-libitum all instances of aggression, dominance and submission with a clear winner-loser outcome, including partner identity.

### Statistical analyses

We used data from experimental tasks and focal observations to run statistical analyses in R (R Core Team, version 4.0.2, 2022). Analyses included the assessment of individuals’ social rank with the Elo system, generalized linear mixed models (GLMM), and social network analyses. First, for each study group, we calculated the rank of each individual with the Elo system, using the EloRating package (version 0.43) in R (Neumann et al. 2011). Elo ranks are decimal values from 0 to 1, which vary depending on the outcome of dyadic agonistic interactions (i.e., all aggressive, dominant and submissive behaviours with a clear winner-loser outcome, see above) and the ranks of the two interacting individuals (Neumann et al. 2011). We observed 303 interactions in wolves (involving 72 of the 91 possible dyads), 68 in AW1 (involving 9 of the15 possible dyads), and 8 in AW2 (involving 2 of the 3 possible dyads). Notably, to establish the dominance hierarchies in each group, it is not necessary to observe agonistic interactions in all possible dyads, as their relative rank can be inferred from the other interactions observed. The individual ranks obtained are listed in Table 2 and have been included in the GLMMs described below.

Second, we ran GLMMs (Baayen et al. 2008) with the glmmTMB package (Brooks et al. 2017) in R (R Core Team, version 4.0.2, 2022). For each of the two tasks, we prepared the dataset by entering one line for each trial and individual that was visible in the trial. The first two full models assessed whether wolves and admixed wolves differed in their reaction to unfamiliar humans. We used a binomial distribution to model whether the probability of showing vigilance (M1) and the probability of spending time in human proximity (i.e. coming closer/remaining at the same distance to the human) in the first ten seconds of the trial (M2) differed between wolves and admixed wolves. In both models we included admixture as test predictor (i.e. wolves vs. admixed wolves). We decided to include admixture rather than group (i.e. wolves, AW1, AW2) as test predictor, because if the introgression of dog genes in the wolf genome leads to important phenotypic changes, these will be evident across study groups despite possible differences in their living conditions and previous life experiences. As controls, we further included individual’s rank (as rank may affect individuals’ reaction to novelty; e.g., Boogert et al. 2006; Crane and Ferrari 2017; Di Bitetti and Janson 2001; Moretti et al. 2015; Stahl et al. 2001) and trial number to control for the fact that neophobia may decrease through trials, as individuals become habituated to the stimuli and set-up. We further included sex and age as controls in the model (to control for the different composition of the study groups in terms of sex and age; see Table 2), and individual identity as random intercept to control for the lack of independencies in the dataset. In M2, however, age had to be removed from the models to avoid collinearity, as suggested by the VIFs > 6. In the human task, only wolves showed fearful and aggressive behaviour within the experimental context, and only admixed wolves explored humans. Therefore, we refrained from running models for these variables.

The next four full models assessed whether wolves and admixed wolves differed in their reaction to novel objects. We used a binomial distribution to model whether the probability of showing vigilance in the first ten seconds of the trial (M3), and the probability of showing fearful behaviour (M4) or exploring the novel object during the trial (M6) differed between wolves and admixed wolves. We further used a beta distribution to model whether the proportion of time spent in proximity to novel objects (M5) differed between wolves and admixed wolves. As above, we entered admixture as test predictor (i.e. wolves vs. admixed wolves), and we included as controls individuals’ rank, trial number, object used (i.e., ball or kettlebell), individual’s sex and age (except for M3, in which age had to be removed from the models to avoid collinearity, as suggested by the VIFs > 6). Finally, we included individual identity as random intercept. In M5, we also included the approximate initial distance of the individual to the object (to account for the fact that closer individuals would have been more likely to move away from the object over the trial), and in M4 and M5 we further included observational effort as offset term (to control for inter-individual differences in the time individuals were visible). This was not necessary in the other models, as all the visible individuals were coded in the first 10 s of the trial (M1–M3), or the response was modelled as a proportion (M6). In the object task, only wolves showed aggressive behaviour within the experimental context (with 7 out of 13 individuals showing aggressive behaviour at least once in the trial), so we refrained from running models for this variable.

All continuous predictors and controls (i.e. rank, age, trial number) were *z*-transformed. We then used likelihood ratio tests to compare each full model containing test predictors, controls, offset terms and random factors to a corresponding null model only containing controls, offset terms and random factors (Dobson and Barnett 2018). If the full model significantly differed from the null model, we used the drop1 function to assess which test predictors and controls of the full model had a significant effect on the response. Full models included no interaction terms, to avoid excessive complexity of the models. We further checked residual diagnostics and multicollinearity using the DHARMa package (Hartig 2022) and the performance package (Lüdecke et al. 2021). We detected no problems in plot residuals and no convergence issues, overdispersion or multicollinearity among predictors and/or controls in any of the models presented (max VIF across all models = 3.73).

Finally, based on the data obtained from the behavioural observations, we calculated the proportion of time that each possible dyad in the group spent in proximity (out of the total time each of the two individuals was observed), and constructed one weighted undirected matrix for each study group. We aimed to prepare similar matrices for the dyadic proportion of time spent in social play and grooming, but as these behaviours were very scant in admixed wolves (see Results), we refrained from running these analyses. For each proximity matrix, we ran social network analyses with the asnipe (version 1.1.10; Farine 2018) and igraph packages (version 1.2.1; Csardi and Nepusz 2006). We extracted weighted densities (i.e., the sum of all the edge weights of the network, divided by the number of all possible edges), which are a common measure of network structure estimating how well-connected the network is (Farine and Whitehead 2015; Farine 2017, 2018; Sosa et al. 2021). Even if weighted densities are mathematically controlled for group size (being divided for the number of all possible edges), they might still differ across groups having different sizes, for instance because in larger groups time constraints reduce the proportion of time that individuals can on average invert in social interactions with each group member (leading to lower densities; see Balasubramanian et al. 2018; Farine and Whitehead 2015). Therefore, for explorative purposes, we repeatedly (i.e. 1000 times) and randomly removed 8 of the 14 individuals from the larger group (W), so that the group size was similar to the one of the admixed groups. We then compared the distribution of these weighted densities to the densities of the two admixed groups, using the median and quantiles of this distribution.

## Results

**Fig. 1:**
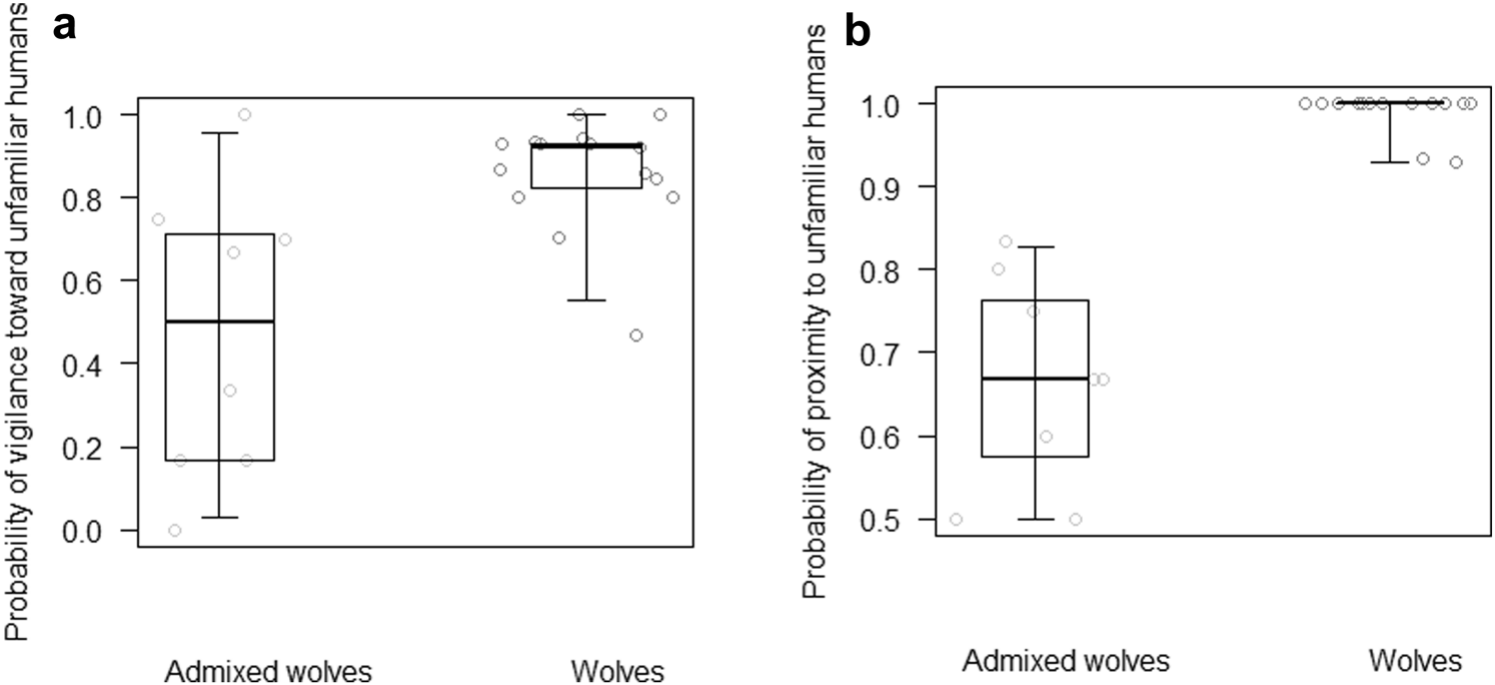
For admixed wolves and non-admixed wolves (i.e. wolves), **a** probability of showing vigilance toward unfamiliar humans, and **b** probability of being in proximity (i.e. remaining at the same distance or coming closer) to unfamiliar humans, as assessed in the first 10 s of the human task trials. Circles represent average values for each individual, in admixed wolves (in light grey) and wolves (in dark grey). The thick lines represent the median values for admixed wolves and wolves, the horizontal ends of the box represent the 75% and 25% quartiles, and the ends of the whiskers represent the 97.5% and 2.5% quartiles

Table 3 shows the average and standard deviation of the responses given by wolves and admixed wolves (also separately for the two study groups) in the object and human tasks (i.e. vigilance, fearful and aggressive behaviour, proximity and exploration). Table 4 reports the results of the GLMMs, including the estimates and confidence intervals of all test predictors and controls. Wolves were more likely to be vigilant towards unfamiliar humans than admixed wolves in the first ten seconds of the human task (M1, GLMM, χ^2^ = 3.90, df = 1, *p* = 0.048; Fig. 1a). Moreover, in the human task only wolves showed fearful and aggressive behaviour during the trial (4 and 10 times, respectively). Wolves were also more likely than admixed wolves to be in proximity to unfamiliar humans in the first ten seconds of the human task (M2, GLMM, χ^2^ = 34.03, df = 1, *p* < 0.001; Fig. 1b). Finally, only admixed wolves showed exploration in the human task, with one individual once approaching within 5 m and sniffing the unfamiliar human during the trial, without showing fearful or aggressive behaviour.

**Table 3:** Mean (± standard deviation) probability of obtaining a given response by admixed wolves vs. non-admixed wolves in captivity (in parentheses, for the two groups separately) in each experimental task (two asterisks denote behaviours significantly differing between admixed and non-admixed wolves, as assessed running generalized linear mixed models, whereas one asterisk denotes behaviours for which no models were run, while the behaviour was observed only in wolves or only in admixed wolves; cf. Table 1)

Response	Wolves	Admixed wolves (AW1/AW2)
Human task		
Probability of being vigilant in the first ten seconds of the trial**	0.86 ± 0.13	0.47 ± 0.33 (0.35 ± 0.28/0.83 ± 0.17)
Probability of fearful behaviour during the trial*	0.02 ± 0.03	0.00 ± 0.00 (0.00 ± 0.00/0.00 ± 0.00)
Probability of aggressive behaviour during the trial*	0.04 ± 0.10	0.00 ± 0.00 (0.00 ± 0.00/0.00 ± 0.00)
Probability of being in proximity to unfamiliar humans in the first ten seconds of the trial**	0.99 ± 0.02	0.66 ± 0.12 (0.69 ± 0.12/0.58 ± 0.08)
Probability of exploring unfamiliar humans during the trial*	0.00 ± 0.00	0.04 ± 0.11 (0.00 ± 0.00/0.17 ± 0.17)
Object task		
Probability of being vigilant in the first ten seconds of the trial**	0.14 ± 0.22	0.67 ± 0.47 (0.75 ± 0.43/0.50 ± 0.50)
Probability of fearful behaviour during the trial	0.40 ± 0.16	0.19 ± 0.22(0.12 ± 0.15/0.31 ± 0.27)
Probability of aggressive behaviour during the trial*	0.15 ± 0.16	0.00 ± 0.00 (0.00 ± 0.00/0.00 ± 0.00)
Proportion of time spent in proximity to novel objects during the trial**	0.54 ± 0.06	0.85 ± 0.11 (0.82 ± 0.10/0.90 ± 0.11)
Probability of exploring novel objects during the trial**	0.29 ± 0.17	0.04 ± 0.11 (0.00 ± 0.00/0.11 ± 0.16)

**Table 4:** Structure of the generalized linear mixed models developed for each experimental test to contrast the behaviour of wolves and admixed wolves in captivity, with estimates, standard deviation (SD), 95% confidence intervals (CIs) and P values

Predictors	Estimate	SD	95% CIs	P value
M1—probability of being vigilant in the first ten seconds of the trial (human task)				
Intercept	0.54	0.62	− 0.68 to 1.76	–
Admixture (wolves)	1.34	0.64	0.09 to 2.60	0.048*
Rank	0.82	0.22	0.38 to 1.25	0.001
Trial number	− 0.33	0.20	− 0.72 to 0.07	0.099
Sex (male)	− 0.39	0.50	− 1.37 to 0.59	0.419
Age	− 0.22	0.30	− 0.81 to 0.37	0.474
M2—probability of being in proximity to unfamiliar humans in the first ten seconds of the trial (human task)				
Intercept	− 0.86	0.84	− 2.51 to 0.79	–
Admixture (wolves)	5.67	1.24	3.23 to 8.10	< 0.001*
Rank	− 0.28	0.24	− 0.76 to 0.20	0.246
Trial number	− 1.17	0.45	− 2.05 to − 0.29	0.009
Sex (male)	2.30	1.09	0.17 to 4.43	0.034
M3—probability of being vigilant in the first ten seconds of the trial (object task)				
Intercept	4.99	2.43	0.22 to 9.77	–
Admixture (wolves)	− 4.24	1.60	− 7.38 to − 1.10	< 0.001*
Rank	− 0.45	0.60	− 1.64 to 0.73	0.446
Trial number	− 1.23	0.74	− 2.69 to 0.23	0.062
Kind of object (kb)	1.33	1.01	− 0.65 to 3.31	0.168
Sex (male)	− 2.34	1.51	− 5.31 to 0.63	0.071
M4—probability of fearful behaviour during the trial (object task)				
Intercept	− 4.97	1.33	− 7.57 to − 2.37	–
Admixture (wolves)	− 1.51	1.35	− 4.15 to 1.13	0.305
Rank	0.42	0.47	− 0.50 to 1.34	0.369
Trial number	0.73	0.40	− 0.04 to 1.51	0.050
Kind of object (kb)	− 5.62	1.17	− 7.91 to − 3.33	< 0.001
Sex (male)	− 1.51	0.80	− 3.07 to 0.06	0.052
Age	0.08	0.74	− 1.38 to 1.54	0.915
M5—proportion of time in proximity to novel objects during the trial (object task)				
Intercept	2.75	0.55	1.68 to 3.83	–
Admixture (wolves)	− 2.38	0.51	− 3.38 to − 1.39	< 0.001*
Rank	− 0.01	0.17	− 0.33 to 0.32	0.957
Trial number	− 0.16	0.12	− 0.40 to 0.08	0.187
Kind of object (kb)	0.70	0.40	− 0.08 to 1.49	0.084
Sex (male)	− 0.46	0.28	− 1.01 to 0.08	0.094
Age	0.21	0.25	− 0.29 to 0.70	0.418
Initial distance from object	1.26	0.21	0.85 to 1.68	< 0.001
M6—probability of exploring novel objects during the trial (object task)				
Intercept	− 10.65	1.90	− 14.38 to − 6.92	–
Admixture (wolves)	4.52	2.26	0.08 to 8.95	0.015*
Rank	1.11	0.73	− 0.31 to 2.54	0.097
Trial number	− 1.43	0.47	− 2.34 to − 0.51	< 0.001
Kind of object (kb)	4.21	1.21	1.85 to 6.57	< 0.001
Sex (male)	− 1.14	1.04	− 3.17 to 0.89	0.248
Age	1.01	0.86	− 0.67 to 2.68	0.224

For each model, controls are in italics and reference categories are in parentheses; kb stands for kettlebell, and the asterisks denote a significant P value for test predictors. Rank, trial number and age were previously *z*-transformed. Variation in the probability of fearful, aggressive and exploratory reactions in the human task, as well as in the probability of aggressive reactions in the object task, could not be tested as these behaviours were observed only in wolves or only in admixed wolves (see text)

**Fig. 2:**
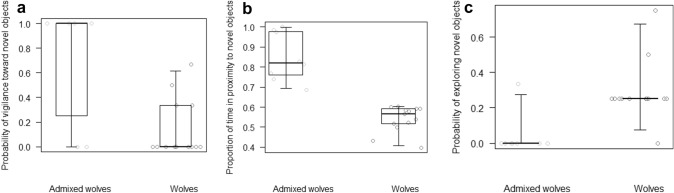
For admixed wolves and non-admixed wolves (i.e. wolves), **a** probability of showing vigilance toward novel objects, **b** proportion of time spent in proximity to novel objects, and **c** probability of exploring novel objects, as assessed **a** in the first 10 s and **b**, **c** over the whole trial of the object task. Circles represent average values for each individual, in admixed wolves (in light grey) and wolves (in dark grey). The thick lines represent the median values for admixed wolves and wolves, the horizontal ends of the box represent the 75% and 25% quartiles, and the ends of the whiskers represent the 97.5% and 2.5% quartiles

Admixed wolves were more likely than wolves to show vigilance in the first ten seconds of the object task (M3, GLMM, χ^2^ = 11.45, df = 1, *p* < 0.001; Fig. 2a). In contrast, we found no differences between wolves and admixed wolves in the probability of showing fearful behaviour toward novel objects (M4, GLMM, χ^2^ = 1.05, df = 1, *p* = 0.305). Moreover, in the object task there were 8 instances of aggressive behaviour, all by wolves. Admixed wolves spent a higher proportion of time than wolves in proximity to novel objects (M5, GLMM, χ^2^ = 20.52, df = 1, *p* < 0.001; Fig. 2b), whereas wolves were more likely than admixed wolves to explore novel objects (M6, GLMM, χ^2^ = 5.94, df = 1, *p* = 0.015; Fig. 2c).

Social network analyses revealed that weighted densities for proximity were relatively low in all study groups (AW1: 0.023; AW2: 0.000; W: 0.019). When repeatedly sampling matrices with 6 individuals from the larger W group, we obtained a distribution of weighted densities with a median of 0.018 (95% quartiles: 0.011–0.028). This exploratory analysis suggests that the networks of AW1 and W were similarly well-connected, as the density of AW1 fell within the 95% quartile range of W. However, only W dyads engaged in grooming (i.e. 9/91 or 9.9% of dyads, vs. 0/18 dyads in admixed wolves). Similarly, play interactions were more common in W dyads (i.e. 18/91 or 19.8% of dyads) than in admixed wolves (i.e. 1/18 or 5.6% of dyads).

## Discussion

In this study, we conducted a first exploratory comparison of captive wolves and admixed wolves. Largely in line with our hypotheses (Table 1), we consistently found behavioural differences in how wolves and admixed wolves reacted to unfamiliar humans and novel objects, and in the cohesiveness of their social networks (Table 1). As predicted (Prediction 1), when exposed to unfamiliar humans in the experimental task, wolves were more vigilant, fearful, and aggressive than admixed wolves, and less likely to interact with humans (i.e., approaching within 5 m and sniffing them). Unexpectedly, however, wolves were also more likely than admixed wolves to spend time in human proximity. When exposed to novel objects, as predicted (Prediction 2), wolves were more aggressive than admixed wolves within the experimental context, less likely to spend time in object proximity, and more likely to interact with objects. Unexpectedly, however, wolves were less vigilant and as fearful as admixed wolves. Finally, in line with our predictions (Prediction 3), we revealed more cohesive social networks in wolves than admixed wolves, although weighted densities for proximity networks were similar in wolves and one group of admixed wolves (AW1).

When exposed to unfamiliar humans, admixed individuals were less vigilant, less fearful and less aggressive than wolves, and they were also more likely to approach humans, without showing fearful or aggressive behaviour. These results are in line with the hypothesis that the introgression of dog genes might trigger behavioural changes in how wolves relate to humans. Comparative studies have indeed provided compelling evidence that dogs are less neophobic than wolves toward humans (e.g., Bentosela et al. 2016; Klinghammer and Goodmann 1987; Range and Marshall-Pescini 2022), likely as a result of the domestication process selecting individuals with higher tolerance and/or attraction to humans (Hare et al. 2002; Li et al. 2013; vonHoldt et al. 2017; vonHoldt and Driscoll 2016). Given the genetic basis of these behaviours (e.g., Salomons et al. 2021; vonHoldt et al. 2017), dog admixture may thus lead to a decrease in wolves’ levels of fear and aggression toward humans, and an increase in the likelihood of interactions, as suggested by our results. In contrast to our predictions, however, wolves were more likely than admixed individuals to remain at the same distance or move closer to unfamiliar humans in the first ten seconds of the human task. In this study, however, proximity to humans did not necessarily reflect increased attraction or tolerance, but it could also result from wolves being more territorial and/or defensive in the presence of unfamiliar humans. Therefore, it is possible that wolves, in this task, perceived unfamiliar humans as a potential danger (as confirmed by the higher occurrence of vigilant, fearful and aggressive behaviour within the experimental context) and were also more likely to maintain proximity, without retreating, as an expression of their territorial behaviour (e.g., Mech 1993; Mech and Boitani 2003). Caution is however needed when interpreting these results, as it is not possible to completely rule out alternative explanations, including differences in the degree of exposure to humans (i.e. although wolves were exposed to humans more often than admixed individuals, early exposure to humans in some admixed individuals might have affected their behaviour toward unfamiliar humans).

When exposed to novel objects, differences between wolves and admixed individuals were less clear-cut. As predicted, admixed wolves showed less aggressive behaviours and were more likely than wolves to be in proximity of novel objects, suggesting a link between dog admixture and lower neophobia. However, admixed individuals were also more vigilant than wolves, and as fearful. Therefore, we only found partial support to the hypothesis that dog admixture is linked to lower object neophobia. These results can be explained in at least two ways. First, it is possible that domestication has selected for individuals that were specifically more tolerant and/or attracted to humans, but not to the human environment (Hare et al. 2002; Li et al. 2013; vonHoldt and Driscoll 2016). If so, the introgression of dog gene variants would lead to clear changes in wolf reaction to humans, as suggested by our results in the human task, but not to changes in their reaction to human artefacts. Second, it is possible that multiple evolutionary pressures contribute to shaping how species react to novel objects. Wolves, in particular, might be more neophobic than dogs (and perhaps admixed wolves), because dog domestication has selected for individuals that are more attracted to humans and their artifacts (Fritts et al. 2003; Hare and Tomasello 2005; Kaulfuβ and Mills 2008), but also because high wolf persecution by humans likely provided selective advantages to more fearful wolves (Fritts et al. 2003; Range and Marshall-Pescini 2022). However, wolves and dogs also differ in terms of the ecological challenges they face: in contrast to dogs, wolves largely live on the preys they hunt, which implies that high neophobia may be detrimental for their survival (see Moretti et al. 2015; Peterson and Ciucci 2003). Moreover, wolves are also more territorial than dogs, and they might thus be more explorative and persistent when interacting with novel objects (Marshall-Pescini et al. 2017; Moretti et al. 2015; Rao et al. 2018), as also confirmed by our object task, in which wolves were more explorative than admixed individuals. Therefore, how admixed wolves react to novel objects might be the result of a complex interplay of multiple factors, as also shown in dogs and wolves (see Range and Marshall-Pescini 2022, for a review). This complex interaction of different factors might thus explain why wolves in our study approached novel objects more cautiously, but also showed less vigilance and more attempts to interact with them (for similar results in dogs and wolves, see Moretti et al. 2015).

Social network analyses suggested higher cohesiveness in wolves than admixed wolves, in terms of grooming and social play, whereas wolf proximity networks were similarly cohesive only to one group of admixed wolves (AW1), the one only including siblings. Grooming, for instance, was only observed in wolves, whereas play and proximity were only observed in wolves and (to a minor extent) in one of the two admixed groups (AW1). These differences between wolves and admixed individuals are in line with studies showing that dogs generally form looser bonds with their conspecifics and are more solitary than wolves (e.g., Beck 1973, 1975; Berman and Dunbar 1983; Daniels 1983; Daniels and Bekoff 1989; Ortolani et al. 2009; Rubin and Beck 1982; see Bonanni and Cafazzo 2014; Cafazzo et al. 2018). Therefore, it is possible that, through the introgression of dog genes, admixed wolves become less likely to engage in affiliative behaviours with other group members, leading to the formation of less cohesive groups. These findings, however, should be taken with caution, as it is not possible to rule out alternative explanations for our results. In our study, for instance, wolves lived in a smaller enclosure than both admixed groups, and this might have favoured a higher rate of encounters and affiliative behaviours in the group. However, this explanation is unlikely, as proximity networks were similar for W and AW1, but social interactions were still more common in admixed dyads. Moreover, as limited space may be a source of stress, affiliative interactions might have been more frequent in wolves than in hybrids as an effective way of reducing stress levels in the group (see Aureli and Smucny 2000; Bonanni et al. 2010). Furthermore, the lack of social interactions in AW2 could have depended on the fact that group members were not all siblings (in contrast to AW1) but came from different litters (although this was also the case for W), and were moved in the new enclosure only shortly before our observations. Therefore, more wolf and admixed groups with similar size, kinship, and enclosures will have to be included to confirm our preliminary findings.

Overall, our study provides a first direct comparison of behavioural traits in admixed and non-admixed wolves, but the results should be taken with caution, due to several important limitations. First, our study sample was limited to only two admixed groups and one wolf group. This implies that the specific characteristics of the study groups (e.g., group size and composition, kin relationship across individuals, previous life experiences, exposure to visitors, time since group establishment) cannot clearly be disentangled from the test predictor (i.e. admixture). To avoid these confounds, future studies should ideally include more groups with different characteristics, although this will not be an easy endeavour, given that groups of admixed wolves are rarely found in captivity. However, it should be noted that most of these confounds cannot alone explain our results. Group size, for instance, was larger in wolves than admixed groups, but this should have led to lower neophobia and lower densities in the social network (e.g., Balasubramanian et al. 2018; Farine and Whitehead 2015), which was not the case in our study. Moreover, behavioural differences between wolves and admixed individuals were mostly in different directions depending on the task (e.g., wolves were more vigilant and fearful in the human task, but not in the object task), suggesting that group size, at best, played a marginal role in explaining these differences. Especially important is the fact that our study subjects were not raised for the purpose of the experiment, so that their living conditions (including group size and composition) and their previous life experiences were not identical. Although all the admixed wolves spent the first weeks of their life with their mothers, for instance, they were then raised by humans in captivity from an early age, and this might have made them less fearful and aggressive to humans, as compared to admixed wolves living in the wild. Therefore, it is not possible to exclude that early experience partially affected the behaviour of our study subjects, and future studies should ideally compare wolves and admixed individuals with strictly controlled life experiences (i.e. having been bred and raised in identical conditions), and/or living in the wild. However, it is unlikely that, in the close future, there will be opportunities to compare wolves and admixed individuals in similar ideal settings, as this raises major ethical concerns. Moreover, it should also be noted that wolves, like admixed individuals, were born and housed in a zoo, where they were regularly exposed to keepers and visitors from an early age, even more frequently than admixed wolves.

A second important limitation of our study is that our results may not be generalizable to all cases of dog-admixture, because different parental dog breeds are partly characterized by different behavioural traits (e.g., Serpell and Duffy 2014). Third, the facilities in which we conducted our studies did not allow individuals to be separated during the tasks, because experimental manipulations could be stressful for the groups. Therefore, it is possible that individuals would show different responses toward novel stimuli when tested alone, because the presence of other group members might facilitate (e.g., social facilitation) and/or hinder (e.g., presence of higher-ranking conspecifics) individuals’ responses. Finally, while our study provided a first assessment of individuals’ reaction to novel stimuli and the cohesiveness of their social networks, differences in other behavioural and cognitive traits can also be expected (e.g., innovation, risk taking, reconciliation patterns), as these traits have been reported to differ between dogs and wolves (Range and Marshall-Pescini 2022).

Despite the above caveats, our study provides a first significant exploration of possible behavioural differences between admixed and non-admixed wolves in captivity. Our results suggest that dog admixture may decrease cohesiveness in wolf social groups, and also explain variation in how wolves react to unfamiliar humans, and partially to novel objects. These findings contribute novel data to the ongoing debate about the possible effects of dog-introgression in wolves, especially with regards to the relationship between humans and wolves, and they may help increasing our understanding of the impact that anthropogenic hybridization has on phenotypic variation in wild population of wolves. If supported by future studies, our findings might also have key implications for conservation. A better understanding of how anthropogenic hybridization, and the extent of admixture, might affect wolf behaviour will contribute to making more informed decisions about the effects and management of admixed individuals in the wild (Donfrancesco et al. 2019). Based on our findings, however, we maintain that by no means it can be assumed that admixed and non-admixed wolves behave similarly. Further studies should investigate the behaviour and ecology of admixed wolves possibly adopting genomic tools to ascertain the genetic origin of behavioural variations in admixed individuals.

## Data Availability

Data will be made available upon reasonable request to the corresponding author.
